# IGF2BP2-modified circular RNA circARHGAP12 promotes cervical cancer progression by interacting m^6^A/FOXM1 manner

**DOI:** 10.1038/s41420-021-00595-w

**Published:** 2021-08-14

**Authors:** Fei Ji, Yang Lu, Shaoyun Chen, Yan Yu, Xiaoling Lin, Yuanfang Zhu, Xin Luo

**Affiliations:** 1 Department of Obstetrics and Gynecology, Shenzhen Baoan Women’s and Children’s Hospital, Jinan University, Shenzhen, 518102 China; 2 The First Clinical Medical College of Jinan University, Guangzhou, 510632 China; 3 Maternal-Fetal Medicine Institute, Shenzhen Baoan Women’s and Children’s Hospital, Jinan University, Shenzhen, 518102 China; 4 Department of Obstetrics and Gynecology, The First Affiliated Hospital of Jinan University, Guangzhou, 510632 China

**Keywords:** Cancer metabolism, Gynaecological cancer

## Abstract

Emerging evidence indicates that circular RNA (circRNA) and N^6^-methyladenosine (m^6^A) play critical roles in cervical cancer. However, the synergistic effect of circRNA and m^6^A on cervical cancer progression is unclear. In the present study, our sequencing data revealed that a novel m^6^A-modified circRNA (circARHGAP12, hsa_circ_0000231) upregulated in the cervical cancer tissue and cells. Interestingly, the m^6^A modification of circARHGAP12 could amplify its enrichment. Functional experiments illustrated that circARHGAP12 promoted the tumor progression of cervical cancer in vivo and vitro. Furthermore, MeRIP-Seq illustrated that there was a remarkable m^6^A site in FOXM1 mRNA. CircARHGAP12 interacted with m^6^A reader IGF2BP2 to combine with FOXM1 mRNA, thereby accelerating the stability of FOXM1 mRNA. In conclusion, we found that circARHGAP12 exerted the oncogenic role in cervical cancer progression through m^6^A-dependent IGF2BP2/FOXM1 pathway. These findings may provide new concepts for cervical cancer biology and pathological physiology.

## Introduction

Cervical cancer is the second most frequent malignant cancer for women worldwide and the third primary cause of cancer-related death in developing countries [1, 2]. Although the increasing application of cervical smear screening and the cervical cancer vaccine make the mortality and morbidity of cervical cancer decreasing, the molecular mechanism underlying cervical cancer is not entirely explicit [3]. In view of this situation, the therapeutic effect for cervical cancer is dissatisfied, we should pay attention to the targeted therapy tracking the pathogenesis.

Circular RNAs (circRNAs) are a group of non-coding RNA without protein-coding ability [4, 5]. More and more researches found that deregulated levels of circRNAs are involved in the initiation and progression of cervical cancer [6, 7]. For example, a representative circRNA circ-ITCH is lowly expressed in cervical cancer tissues and cells. Overexpression of circ-ITCH significantly suppresses the cellular proliferation, migration/invasion through sponging micRNA-93-5p, and regulating the expression of FOXK2 [8]. Moreover, upregulated circRNA hsa_circRNA_101996 serves as a miR-8075 sponge to target TPX2 in cervical cancer, thereby promoting cervical cancer proliferation and invasion [9]. Therefore, these data suggests that circRNA might function as an oncogenic or anticancer element.

N^6^-methyladenosine (m^6^A) is the most abundant RNA modification occurring in eukaryotic mRNAs [10]. m^6^A modification is a dynamic and reversible process mediated by two types of catalytic enzymes, including m^6^A writer (METTL3, METTL14, KIAA1429, and WTAP) and eraser (FTO, ALKBH5) [11–13]. Besides, another m^6^A enzymes (m^6^A readers) could regulate the fate of m^6^A-containing mRNAs, including nuclear, export, mRNA stability, and mRNA translation. It has been identified that m^6^A could regulate tumorigenic progression. For example, m^6^A methyltransferase METTL3 is significantly upregulated in cervical cancer tissue and cells and promotes the proliferation, Warburg effect (aerobic glycolysis) through targeting the 3′-UTR of HK2 mRNA to enhance HK2 stability [14]. Moreover, YTHDF1/eEF-2 complex and IGF2BP3 positively promote the translation elongation and mRNA stability of pyruvate dehydrogenase kinase 4 (PDK4) to regulate glycolysis of cervical cancer cells [15]. Thus, these findings suggest that m^6^A might act as an essential regulator in cervical cancer progression.

In this study, we performed the circRNA microarray analysis in cervical cancer and results demonstrated that a novel circARHGAP12 (hsa_circ_0000231) was significantly upregulated in the cervical cancer tissue and cells. CircARHGAP12 was 794 bp length and generated from exon 3 and exon 2 in the ARHGAP12 gene. Our research found that circARHGAP12 could enhance the stability of FOXM1 mRNA via binding with IGF2BP2. Thus, our data suggest that circARHGAP12/m^6^A/IGF2BP2/FOXM1 axis in the cervical cancer tumorigenesis.

## Results

### circARHGAP12 was an unfavorable circRNA for cervical cancer

**Fig. 1:**
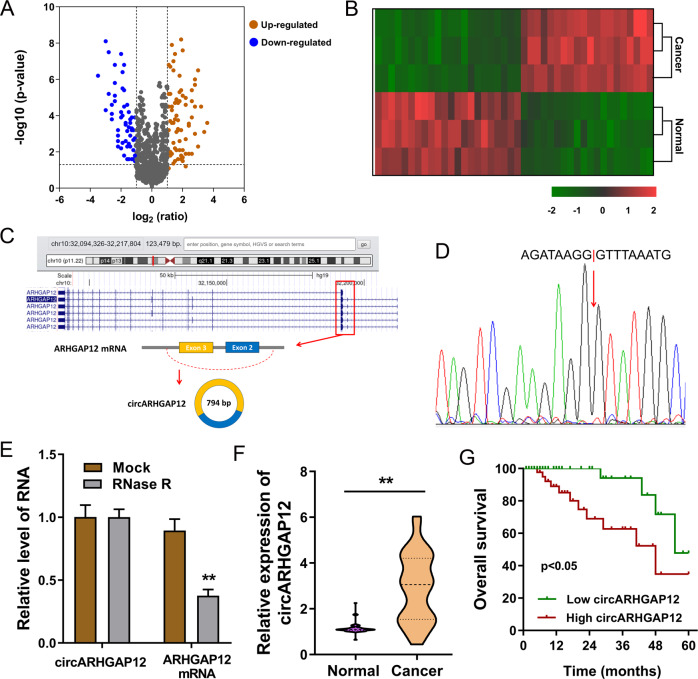
circARHGAP12 was an unfavorable circRNA for cervical cancer. **A** Volcano plot of circRNA microarray analysis revealed the des-regulated circRNAs within the cervical cancer tissue and adjacent normal tissue. **B** Heatmap of circRNA microarray analysis. **C** Schematic diagram demonstrated the generation of circARHGAP12 (hsa_circ_0000231, 796-bp) from exon-3 and exon-2 of ARHGAP12 gene through back splicing. **D** Sanger sequencing revealed the conjunction sites of circARHGAP12 back splicing. **E** RNA persistence testing using RNase R showed the expression of circular transcript (circARHGAP12) and linear mRNA (ARHGAP12 mRNA) detected using RT-PCR. **F** In the collected cervical cancer specimens’ group, circARHGAP12 expression was analyzed using RT-PCR. **G** Survival analysis using the Kaplan–Meier survival curve and the log-rank test demonstrated the survival rate of patients with higher or lower circARHGAP12 levels. P values were calculated by Student’s *t*-test. **p < 0.01.

Using the circRNA microarray analysis, we found that there were hundreds of circRNAs varied within the cervical cancer tissue and adjacent normal tissue (Fig. 1A, B). One of the new molecules that attracted our attention was circARHGAP12 (hsa_circ_0000231, 796-bp). CircARHGAP12 was generated from the exon-3 and exon-2 of the ARHGAP12 gene through back splicing (Fig. 1C). Sanger sequencing found that the conjunction sites of circARHGAP12 back splicing was in line with the prediction (Fig. 1D). In the RNA persistence testing using RNase R or control group, RT-PCR analysis found that the circular transcript, rather than linear mRNA, was much resistant to enzymatic digestion (Fig. 1E). In the collected cervical cancer specimens’ group, we found that the expression of circARHGAP12 was much higher as compared to the adjacent normal tissue (Fig. 1F and Table 1). Survival analysis using Kaplan–Meier survival curve and the log-rank test demonstrated that the survival rate of patients with higher circARHGAP12 level was lower than the lower patients with statistical significance (Fig. 1G). These results are consistent with the finding that circARHGAP12 was an unfavorable circRNA for cervical cancer.

**Table 1:** The correlations between circARHGAP12 and cervical cancer patients’ clinicopathological characteristics.

	*N* (48)	circARHGAP12		*p*
		Low (24)	High (24)	
Age				
<45 year	22	10	12	0.415
≥45 year	26	14	12	
Tumor size				
<4 cm	21	14	7	0.013*
≥4 cm	27	10	17	
FIGO stages				
I/II	25	12	13	0.247
III/IV	23	12	11	
Tumor differentiation				
Well/moderate	30	11	19	0.153
Poor	18	13	5	
Lymph node metastasis				
Positive	17	10	7	0.139
Negative	31	14	17	

*indicate the p < 0.05.

### m^6^A modification of circARHGAP12 could increase its stability

**Fig. 2:**
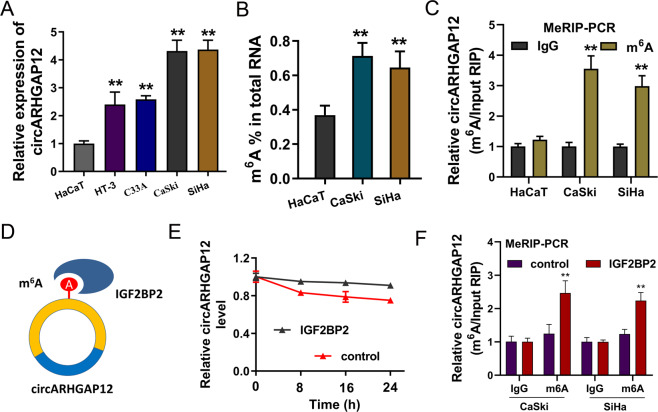
m^6^A modification of circARHGAP12 could increase its stability. **A** RT-PCR showed the circARHGAP12 expression in the cervical cancer cells (HT-3, CaSki, C33A, and SiHa). **B** m^6^A quantitative analysis detected the m^6^A enrichment in the cervical cancer cells (CaSki and SiHa) and normal cells (HaCaT). **C** MeRIP-PCR detected the circARHGAP12 enrichment in anti-m^6^A immunoprecipitation in cervical cancer (CaSki and SiHa) and normal cells (HaCaT). **D** Schematic diagram displayed the interaction within circARHGAP12 and IGF2BP2. **E** RNA stability assay illustrated the circARHGAP12 level in the IGF2BP2 overexpression group and control group. **F** MeRIP-PCR assay measured the circARHGAP12 expression in cervical cancer (CaSki, SiHa) transfected with IGF2BP2 overexpression. Experiments were performed in triplicate. *P* values were calculated by Student’s *t*-test. **p < 0.01.

As shown in the figure, the expression of circARHGAP12 was detected using RT-PCR, and data showed that circARHGAP12 expression was upregulated in the cervical cancer cells (Fig. 2A). The m^6^A enrichment in the cervical cancer cells was higher than the normal cells (Fig. 2B). MeRIP-PCR found that the circARHGAP12 enrichment was higher in the anti-m^6^A immunoprecipitation in cervical cancer (CaSki, SiHa) (Fig. 2C). We found that circARHGAP12 could interact with the specific m^6^A reader IGF2BP2 (Fig. 2D). RNA stability assay illustrated that, in the IGF2BP2 overexpression group, the circARHGAP12 level was slightly higher than the control group (Fig. 2E). Moreover, MeRIP-PCR assay data revealed that the IGF2BP2 overexpression promoted the circARHGAP12 expression in cervical cancer (CaSki, SiHa) (Fig. 2F). Thus, these findings suggested that m^6^A-modified circARHGAP12 could increase its stability.

### circARHGAP12 promoted the tumorigenesis of cervical cancer

**Fig. 3:**
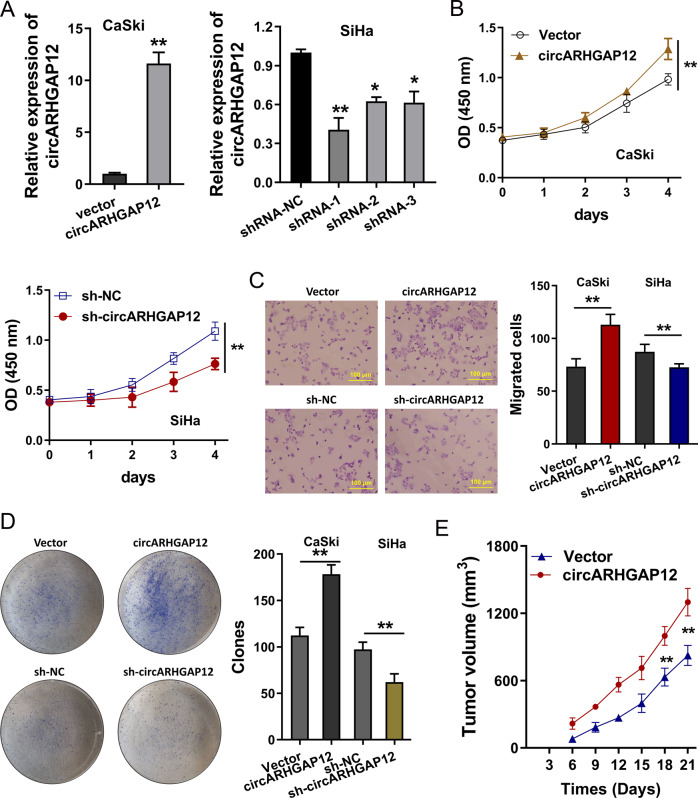
circARHGAP12 promoted the tumorigenesis of cervical cancer. **A** Overexpression and knockdown of circARHGAP12 were constructed using stable transfection. Expression of circARHGAP12 was detected using RT-qPCR. **B** CCK-8 proliferation assay demonstrated the proliferative ability of cervical cancer cells (CaSki and SiHa) respectively transfected with circARHGAP12 knockdown (sh-circARHGAP12) and circARHGAP12 overexpression (circARHGAP12). **C** Transwell migration assay illustrated the migration of cervical cancer cells (CaSki and SiHa) transfected with circARHGAP12 knockdown (sh-circARHGAP12) and circARHGAP12 overexpression (circARHGAP12). **D** Colony formation assays displayed the colony formation of cervical cancer cells. **E** In vivo mice assay found the tumor growth in mice injected of circARHGAP12 overexpression cells (CaSki cells). Experiments were performed in triplicate. *P* values were calculated by Student’s *t*-test. **p < 0.01.

To address whether circARHGAP12 could regulate the tumorigenesis of cervical cancer, the overexpression and knockdown of circARHGAP12 were constructed using stable transfection (Fig. 3A). CCK-8 proliferation assay demonstrated that circARHGAP12 overexpression accelerated the proliferative ability of cervical cancer cells (CaSki), and the knockdown of circARHGAP12 repressed the proliferation of cervical cancer cells (SiHa) (Fig. 3B). Transwell migration assay illustrated that circARHGAP12 promoted the migration of cervical cancer cells (CaSki), and the knockdown of circARHGAP12 inhibited the migration of cervical cancer cells (SiHa) (Fig. 3C). Colony formation assays displayed that circARHGAP12 overexpression enhanced the colony formation and knockdown of circARHGAP12 restrained the clones of cervical cancer cells (Fig. 3D). In vivo mice assay found that the transfection of circARHGAP12 overexpression (CaSki cells) could promote the in vivo tumor growth (Fig. 3E). In summary, these data suggested that circARHGAP12 promoted the tumorigenesis of cervical cancer.

### circARHGAP12 interacted with m^6^A reader IGF2BP2

**Fig. 4:**
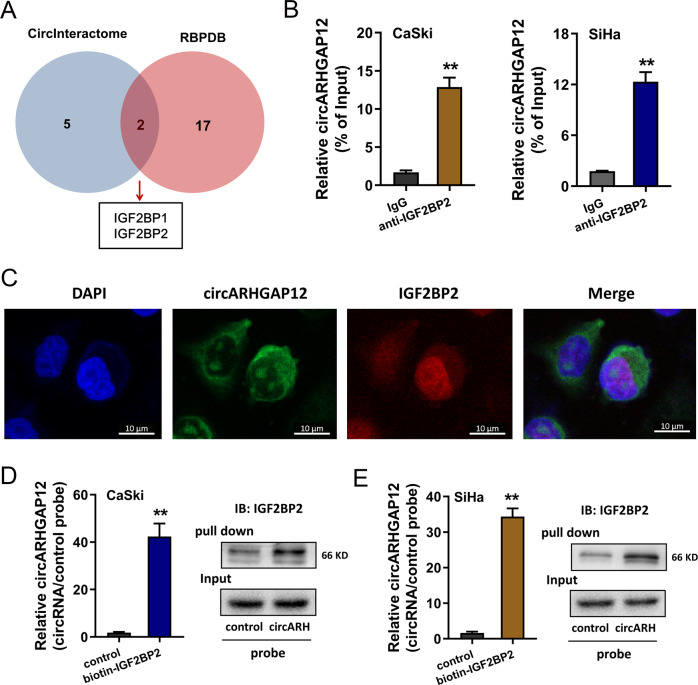
circARHGAP12 interacted with m^6^A reader IGF2BP2. **A** Online bioinformatics tool (https://circinteractome.nia.nih.gov/, http://rbpdb.ccbr.utoronto.ca/) found that circARHGAP12 might interact with the RNA binding protein (RBP) IGF2BP1 and IGF2BP2. **B** RBP immunoprecipitation (RIP) assay following qPCR demonstrated the enrichment of circARHGAP12 in the anti-IGF2BP2 or IgG antibody group (CaSki, SiHa). **C** Fluorescence in situ hybridization (FISH) assay illustrated the distribution of circARHGAP12 and IGF2BP2 in cervical cancer (CaSki) cells. CircARHGAP12 was labeled with the FAM (green) and IGF2BP2 was labeled with the cy3 (red). **D**, **E** Biotin-labeled RNA pull-down was performed using circARHGAP12 probe and control probe. The enrichment of circARHGAP12 was measured using RT-qPCR and the level of IGF2BP2 protein was detected using western blot assay. Experiments were performed in triplicate. *P* values were calculated by Student’s *t*-test. **p < 0.01.

With the help of bioinformatics prediction, we found that circARHGAP12 might interact with the RNA binding protein (RBP) IGF2BP1 and IGF2BP2 (https://circinteractome.nia.nih.gov/, http://rbpdb.ccbr.utoronto.ca/) (Fig. 4A). RBP immunoprecipitation (RIP) assay demonstrated that circARHGAP12 was enriched in the anti-IGF2BP2 antibody group, suggesting the molecular interaction within circARHGAP12 and IGF2BP2 (Fig. 4B). Fluorescence in situ hybridization (FISH) assay illustrated that circARHGAP12 distributed in the cytoplasm of cervical cancer (CaSki) cells, and IGF2BP2 disperse both in the nucleus and cytoplasm (Fig. 4C). Moreover, a pull-down assay was performed and RT-qPCR results found that the enrichment of circARHGAP12 was prominently enriched in the circARHGAP12 probe group as compared to the control probe group (Fig. 4D, E left). Besides, the western blot assay found that IGF2BP2 protein was enriched in the circARHGAP12 probe group (Fig. 4D, E right). Taken together, these findings concluded that circARHGAP12 interacted with m^6^A reader IGF2BP2.

### circARHGAP12/IGF2BP2 enhanced the stability of FOXM1 mRNA

**Fig. 5:**
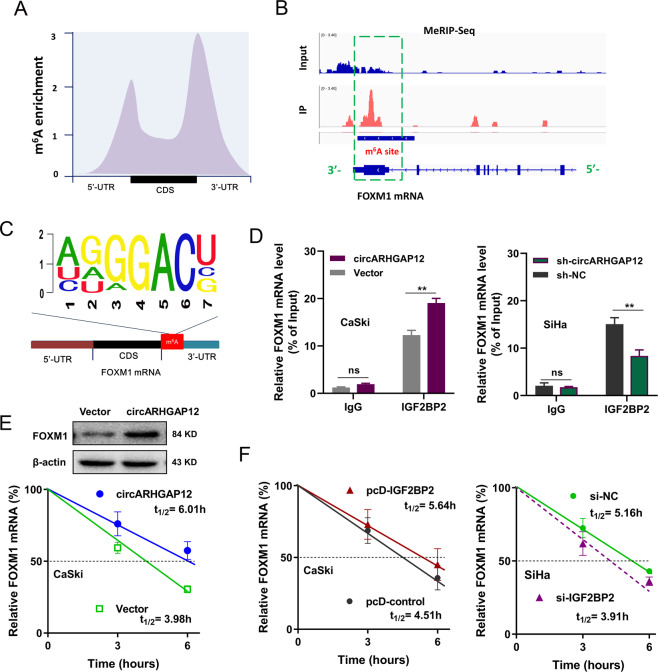
circARHGAP12/IGF2BP2 enhanced the stability of FOXM1 mRNA. **A** MeRIP-Seq was performed to discover the potential m^6^A modification profile in the cervical cancer cells. m^6^A sites were displayed in 5′-UTR, 3′-UTR, and CDS. **B** Schematic diagram based on MeRIP-Seq showed the remarkable m^6^A modification site in the 3′-UTR of FOXM1 mRNA. **C** Symbol showed the m^6^A motif in the FOXM1 mRNA (GGACU). **D** RBP immunoprecipitation (RIP)-qPCR assay demonstrated the FOXM1 mRNA enrichment precipitated by anti-IGF2BP2 antibody in CaSki or SiHa cells transfected with circARHGAP12 (plasmids) overexpression or circARHGAP12 knockdown (sh-circARHGAP12). IgG acted as the blank control. **E** RNA stability assay and western blot assay demonstrated the FOXM1 mRNA and protein level in CaSki cells transfected with circARHGAP12 overexpression. **F** RNA stability assay showed the FOXM1 mRNA expression in CaSki cells transfected with IGF2BP2 overexpression (pcD-IGF2BP2), or in SiHa cells transfected with IGF2BP2 silencing (si-IGF2BP2). Experiments were performed in triplicate. *P* values were calculated by Student’s *t*-test. **p < 0.01.

MeRIP-Seq was performed to discover the potential m^6^A modification profile in the cervical cancer cells (Fig. 5A). Sequencing data demonstrated that the m^6^A sites’ enrichment was displayed in the connection point of 5′-UTR or 3′-UTR and CDS. Evidences had revealed the critical role of FOXM1 in cervical cancer. Coincidently, MeRIP-Seq found that there was a remarkable m^6^A modification site in the 3′-UTR of FOXM1 mRNA (Fig. 5B). The m^6^A motif in the FOXM1 mRNA was found to be GGACU (Fig. 5C). RIP qPCR assay demonstrated that circARHGAP12 overexpression could improve the FOXM1 mRNA enrichment precipitated by anti-IGF2BP2 antibody in the CaSki cells, and circARHGAP12 knockdown reduced the FOXM1 mRNA enrichment precipitated by anti-IGF2BP2 antibody in the SiHa cells (Fig. 5D). RNA stability assay and western blot assay revealed that circARHGAP12 overexpression promoted the FOXM1 mRNA stability (Fig. 5E down) and FOXM1 protein level (Fig. 5E up). Moreover, RNA stability assay indicated that IGF2BP2 overexpression enhanced the FOXM1 mRNA stability (Fig. 5F left), and IGF2BP2 silencing reduced the FOXM1 mRNA stability (Fig. 5F Right). In conclusion, the above data suggested that circARHGAP12/IGF2BP2 enhanced the stability of FOXM1 mRNA.

## Discussion

Cervical cancer acts as one of the most common gynecologic cancers, and increasing evidence suggests the emerging role of circRNAs in cervical cancer tumorigenesis [16, 17]. Moreover, the regulation of m^6^A in cervical cancer catches researchers’ attention. Here, we focused on the function of m^6^A-circRNAs on cervical cancer.

With the aid of circRNA high-throughput sequencing, we found that a novel circRNA circARHGAP12 was upregulated in the cervical cancer tissue and adjacent normal tissue. CircARHGAP12 (hsa_circ_0000231, 794 bp) was generated from the exon 3 and exon 2 of the ARHGAP12 gene. Interestingly, there was an m^6^A modification site in exon 3, indicating the potential m^6^A-circRNA interaction in the circARHGAP12. The special m^6^A modification in the circARHGAP12 clue that the biogenesis or biological characteristic of circARHGAP12 may be varied due to the m^6^A. Subsequently, we discovered that m^6^A reader IGF2BP2 could recognize the m^6^A site in circARHGAP12 and meanwhile enhance its enrichment. Existing researches revealed that m^6^A reader could specifically combine with circRNA to regulate the circRNA fate. For instance, in human immunity, m^6^A modification receded the immune gene activation and adjuvant activity, and m^6^A reader YTHDF2 is essential for suppression of innate immunity through sequestering the m^6^A-circRNA [18]. Another m^6^A reader YTHDC1 connects with m^6^A-modified circ-ARL3, which sponges miR-1305 and thereby promotes HBV+ HCC progression [19]. Therefore, these evidences clue that m^6^A modification could regulate the function of circRNAs.

Functionally, the biofunctional assays found that circARHGAP12 could promote the proliferation and migration of cervical cancer cells (CaSki, SiHa). Besides, in vivo experiments demonstrated that circARHGAP12 accelerated the tumor growth of cervical cancer cells. In the enormous number of circRNAs, circARHGAP12 is a novel identified circRNAs and its functions have been described. For example, in DOX-induced cardiotoxicity, circArhgap12 is upregulated in the mouse heart tissue, and circArhgap12 could sponge miR-135a-5p to regulate ADCY1 mRNA in rat primary cardiomyocytes [20]. In nasopharyngeal carcinoma, circARHGAP12 is significantly upregulated and regulates the expression of cytoskeletal remodeling-related proteins (EZR, TPM3, and RhoA) through directly binding the mRNA 3′-UTR and promoting its stability via EZR/TPM3/RhoA complex [21]. Thus, these data demonstrates the biological role of circARHGAP12.

**Fig. 6:**
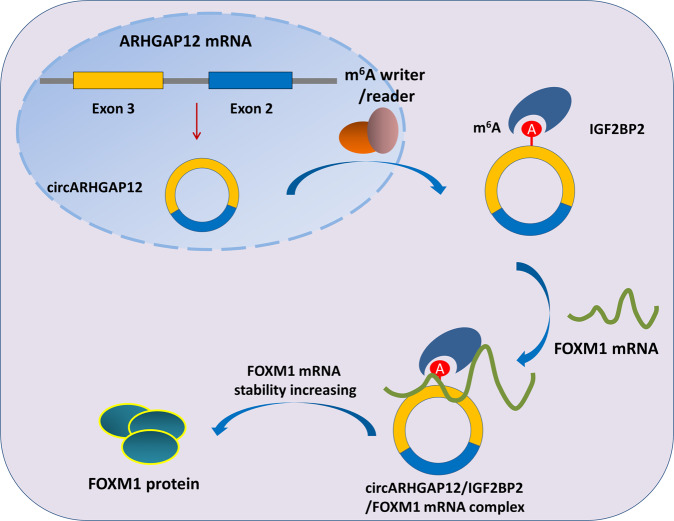
m^6^A-modified circARHGAP12 promotes cervical cancer progression by enhancing FOXM1 mRNA stability via interacting IGF2BP2.

Subsequently, we found that circARHGAP12 interacted with m^6^A reader IGF2BP2 through m^6^A site in the exon-3, one of the elements of circARHGAP12. Subcellular co-localization assay found that circARHGAP12 and IGF2BP2 were collectively located in the cytoplasm. Based on the MeRIP-Seq data, we found that there was a remarkable m^6^A modification site in the 3′-UTR of FOXM1 mRNA. CircARHGAP12 improves the FOXM1 mRNA enrichment precipitated by an anti-IGF2BP2 antibody. In other words, circARHGAP12 interacts with IGF2BP2 to combine with FOXM1, forming circARHGAP12/IGF2BP2/FOXM1 complex (Fig. 6). In cervical cancer, FOXM1 was found to be an oncogene [22]. Thus, this regulation pathway might reveal the oncogenic route for cervical cancer.

Collectively, these findings provide robust evidence that novel circRNA circARHGAP12 acts as an oncogene in cervical cancer tumorigenesis. m^6^A-modified circARHGAP12 interacts with IGF2BP2 to enhance FOXM1 mRNA stability, forming circARHGAP12/IGF2BP2/FOXM1 complex, thereby promoting the proliferation and migration of cervical cancer cells. These achievements might provide solving ideas for the targeted therapy with a view to the m^6^A-circRNA approach.

## Materials and methods

### Clinical patients’ tissue specimen collection

The cervical cancer patients’ tissue specimens (forty-eight cases) were recruited during the surgery. All the volunteers had been informed of the clinical recruitment and understood the experimental procedure. Informed consent was acquired from every patient. Cancer tissue and paired adjacent non-tumor tissues were collected and reserved under −80 °C. The procedures were approved by the Ethics Committee of Shenzhen Baoan Women’s and Children’s Hospital.

### Cervical cancer cells and transfection

The human epidermal cell (HaCaT) and cervical cancer cell lines (HT-3, CaSki, C33A, and SiHa) were provided from the Chinese Academy of Sciences Cell Bank (Shanghai, China). Cells were cultured in RPMI 1640 medium (Invitrogen, Carlsbad, CA) with 10% fetal bovine serum (FBS), 100 μg/mL streptomycin, and 100 U/mL penicillin G at 37 °C in a 5% CO_2_ atmosphere. Lentivirus plasmids of shRNA were constructed by Genechem Company (Shanghai, China) and packaged using pMD2.G and psPAX2 (Addgene, Cambridge, MA) into cell. After transfection, virus supernatants were concentrated to infect cervical cancer cells by polybrene (8 μg/mL, Sigma, MO). Three days later, cells were treated with puromycin (2 μg/mL, Sigma) for 7 days. For circARHGAP12 overexpression, RNA was amplified from reverse-transcribed cDNA and the PCR products were cloned into the pZW1-FCS-circRNA vector (Addgene).

### Quantitative real-time polymerase chain reaction (qRT-PCR)

The total RNA was isolated from cervical cancer cells or tissue samples using Trizol reagent. Total RNA (0.5 μg) was used for reverse transcription for cDNA. Reverse transcription was performed by HiScript Q RT-SuperMix for qPCR (Vazyme, China). PCR was performed using SYBR Green PCR kit (TaKaRa, Dalian, China) on Applied Biosystems 7300 detection system (Applied Biosystems) with triplicate times. All these primers were listed in Supplementary Table S1.

### m^6^A quantitative analysis

Firstly, the total RNA was extracted from cervical cancer cells using TRIzol following the manufacturer’s protocol. The global m^6^A level in total mRNA was detected through the colorimetric EpiQuik m^6^A RNA Methylation Quantification Kit (Epigentek) according to the manufacturer’s instructions. The m^6^A enrichment was colorimetrically detected using the 450 nm absorbance.

### RNase R and actinomycin D treatment

For RNase R treatment assay, 2 mg of total RNA was incubated with or without 5 U/μg RNase R (Epicentre Technologies, Madison WI, USA) at 37 °C for 30 min. Then, RNA was purified using RNeasy MinElute Cleaning Kit (Qiagen) and subsequently calculated by qRT-PCR. For Actinomycin D (Act D) treatment assay, cells (5 × 10^5^ cells/well) were seeded in six-well plates and then exposed to 2 μg/ml Actinomycin D (Sigma). At indicated time points, cells were collected and the RNA stability was analyzed using qRT-PCR normalizing to mock treatment group.

### Methylated RNA immunoprecipitation PCR (MeRIP-PCR)

For the quantitative analysis of m^6^A-modified mRNA, MeRIP-PCR was performed. After the isolation of mRNA, anti-m^6^A antibody and anti-IgG (Cell Signaling Technology) were conjugated with protein A/G magnetic beads in IP buffer (140 mM NaCl, pH 7.5 20 mM Tris, 2 mM EDTA, 1% NP-40, RNase inhibitor, and protease inhibitor) at 4 °C overnight. After incubation, the RNA-beads complex was eluted using elution buffer. Lastly, the RNA enrichment was identified using the qRT-PCR assay calculating the 2^-ΔΔCt^ of eluate relative to the input sample.

### Proliferation assay

Proliferation ability was detected using CCK-8 assay and colony formation assay. For the CCK-8 assay, the transfected cervical cancer cells were added with a cell counting kit-8 (CCK-8) reagent (Dojindo, Japan, 10 mL/well). The optical density (OD) value was detected at 450 nm by an enzyme-mark reader. For colony formation assay, the transfected cervical cancer cells were seeded in the fresh six-well plate in 1640 medium containing 10% FBS. After 14 days, cells were fixed with methanol and stained with crystal violet (0.1%). Lastly, colonies were manually counted.

### Western blot analysis

Total protein in cells was extracted using radioimmunoprecipitation assay (RIPA) buffer containing PMSF (phenylmethanesulfonyl fluoride). After centrifugation (10 min, 4 °C, 25,764x*g*), the supernatant was collected. The concentration of protein in the sample was detected using bicinchoninic acid (BCA) kit and then adjusted by deionized water. SDS-PAGE (10%) was configured for protein separation and then transferred to PVDF (polyvinylidene fluoride) membrane (Millipore). The membranes were administrated with 5% skimmed milk for 2 h and then incubated with primary antibody anti-FOXM1 (1:1000, Abcam, ab207298), rabbit anti-IGF2BP2 (1:1000, Abcam, ab124930), β-actin (1:2000, ab8226, Abcam) at 4 °C overnight. After being washed with PBS tween-20 (PBST) three times, the membrane was immersed in enhanced chemiluminescence (ECL) reaction solution for images exposure.

### Fluorescence in situ hybridization (FISH)

The subcellular location of circARHGAP12 and IGF2BP2 was detected using the FISH assay. In brief, FAM-labeled circARHGAP12 probes, Cy3-labeled IGF2BP2 probes, and DAPI-labeled probes were provided by Genepharma (Shanghai, China). Nuclei was stained by 4,6-diamidino-2-phenylindole (DAPI). The FISH assay was performed using a fluorescent in situ hybridization kit (Genepharma) according to the manufacturer’s protocol. Images were captured with a confocal microscope (Olympus).

### RNA immunoprecipitation (RIP) assay

RIP assay was performed for the RNA–protein interaction using MagnaRIP RNA-Binding Protein Immunoprecipitation Kit (Millipore, MA, USA) according to the manufacturer’s instructions. In brief, cell lysates were extracted from cervical cancer cells. RNA were incubated with antibody-coated buffer containing beads (anti-IGF2BP2, control rabbit IgG, Abcam) at 4 °C overnight. The conjugated precipitation was eluted and detected using qRT-PCR analysis.

### RNA pull-down assay

Biotin-labeled circARHGAP12 probe RNA pull-down assay was synthesized by Genepharma (Shanghai, China). Cervical cancer cells were lysed and incubated with specific probes and streptavidin-coated magnetic beads in a lysis buffer. The biotin-labeled RNA complex was pulled down and the beads were washed and purified. Then, the enrichment of circARHGAP12 was analyzed by qRT-PCR, and the retrieved IGF2BP2 protein was detected using a western blot.

### Mice xenograft model assay

In vivo animal experiments were approved by the Ethics Committee of Shenzhen Baoan Women’s and Children’s Hospital. Ten BALB/c nude mice (male, 15 g) were purchased from the SLAC Laboratory Animal Center (Shanghai, China). CircARHGAP12-stable knockdown cervical cancer cells (100 μL PBS containing 5 × 10^6^ cells) were subcutaneously injected into the lateral flank of BALB/c nude mice. The length and width were measured every 3 days to calculate the tumor volume. Mice were sacrificed after 4 weeks and the tumor was weighed. The injection was randomly performed.

### Statistical analysis

The statistical analysis was performed by GraphPad Prism v7.0 software. All functional experiments were performed for triplicate times and data was displayed as means ± SD (standard deviation). Student’s *t*-test and ANOVA were used to compare the statistical significance within two groups and differences between groups. Among them, *p* < 0.05 or *p* < 0.01 was considered statistical significant.

## Supplementary information

Table S1
