# Supplementary material for: IGF2BP2-modified circular RNA circARHGAP12 promotes cervical cancer progression by interacting m6A/FOXM1 manner
Source: Cell Death Discov. 2021 Aug 14;7:215. doi: 10.1038/s41420-021-00595-w (PMC8364552; doi:10.1038/s41420-021-00595-w)
Supplement: Supplementary file 1 — Table S1 [file 41420_2021_595_MOESM1_ESM.docx]

**Supplement Table 1**. Primers sequences for qRT-PCR and sequences of shRNA.

|  | Sequences |
| --- | --- |
| circARHGAP12 | forward, 5’-ATCTTGTGATTCCGCAGGAG-3’  reverse, 5’-ATGGCTTTATGGCTTGTTGG-3’ |
| sh-circARHGAP12-1 | 5’-ACTGAACAGATAAGGGTTTAA-3’ |
| sh-circARHGAP12-2 | 5’-CTGAACAGATAAGGGTTTAAA-3’ |
| sh-circARHGAP12-3 | 5’-CACTGAACAGATAAGGGTTTA-3’ |
| circARHGAP12 probe | 5’-TCCTCGGTCTGGAACCTTTGTGAATTTGGCATGT-3’ |
| FOXM1 | forward, 5’-CGTCGGCCACTGATTCTCAAA-3'  reverse, 5'-GGCAGGGGATCTCTTAGGTTC-3’ |
| IGF2BP2 | forward, 5’-AGTGGAATTGCATGGGAAAATCA-3’  reverse, 3’-CAACGGCGGTTTCTGTGTC-5’ |
| GAPDH | forward, 5’-GGTATGACAACGAATTTGGC-3’  reverse, 5’-GAGCACAGGGTACTTTATTG-3’ |
